# Exploring factors associated with company employee involvement in physical activities with older psychiatric outpatients: a qualitative study

**DOI:** 10.3389/fpsyt.2025.1619253

**Published:** 2026-01-12

**Authors:** Kristel van Kraanen, Jakobus Smit, Barbara Sassen, Jeroen Deenik, Anja de Kruif, Stella Rijks, Annemiek de Kock, Didi Rhebergen

**Affiliations:** 1 GGZ Centraal, Amersfoort, Netherlands; 2 University of Applied Sciences Utrecht, Utrecht, Netherlands; 3 Amsterdam University Medical Center, Amsterdam, Netherlands

**Keywords:** mental health, older adults, company involvement, employee participation, volunteer-based support, stigma

## Abstract

**Background:**

With ageing societies, the number of older persons with mental health problems are increasing the coming decades. Physical health and loneliness are well known risk factors. Effective interventions, promoting social interaction, are hampered by stigma associated with mental health. This is further compounded by a global mental healthcare and demand gap, highlighting the need for volunteer-based support in addressing the needs of older mental health patients.

**Aim:**

This study aims to explore factors associated with company employee participation in a walking project designed for psychiatric outpatients aged 60 and older.

**Methods:**

A qualitative design was employed. For a diverse representation of perspectives on the topic, contact was sought with HR representatives, vitality managers or directors from 250 companies (profit and nonpofit) with different ownership structures, management styles, and decision-making processes. Data were collected through semi-structured interviews and analyzed using the Constant Comparative Method with a thematic approach via Atlas.ti. Analysis occurred in six phases: data preparation, familiarization, finalization, open coding, axial coding, and selective coding.

**Findings:**

13 of 250 companies engaged in interviews. Respondents viewed the project positively, but highlighted significant barriers to structural volunteer engagement, related to views on volunteerwork, preconditions, and employee-, patient-, and company-related factors. A pilot project with motivated employees is recommended by respondents, aligning initiatives with Corporate Social Responsibility (CSR) goals.

**Conclusion:**

This study highlights the complexity of company involvement within mental health care. By adressing stigma reduction and employee engagement, companies can contribute to a more inclusive and compassionate society.

## 1 Introduction

The aging population is a well-documented global phenomenon. It was estimated that by 2050, 1.5 billion people worldwide will be older than 65, equivalent to one in six people (1). As individuals age, mental health disorders become increasingly prevalent. It was demonstrated that one in three adults (aged 65 to 84 years) in Europe experienced a mental health disorder within the previous year (2), with depression ranking as fourth leading cause of disability in individuals aged 60 and over (3).

Mental health disorders significantly impact well-being of older adults and their families, with complex, multifaceted causes more common than not (4). Factors such as socioeconomic status, physical health, and loneliness often contribute to the onset and persistence of these conditions (5–7). People experiencing mental health problems often struggle to form and maintain social connections, resulting in smaller and less diverse social networks, typically involving only peers, family members, and healthcare professionals (8). Interventions aimed at increasing physical activity or reducing loneliness have shown to improve well-being, aid in the prevention and recovery from psychiatric disorders, and benefit all ages (9–16). Despite the effectiveness of interventions promoting social interaction, older psychiatric patients often face stigmatization (public stigma, i.e., negative attitudes or prejudices held by others), which hamper formation of new social connections crucial for mental health (17).

While prevalence of mental health disorders among older adults and the challenges in addressing their needs are already significant, this is further compounded by the substantial gap between the global demand for mental health care and its supply (18, 19), highlighting the urgent need for innovative solutions. The World Health Organization emphasizes that healthy ageing and long-term care require partnerships between governments, families, communities, health care providers and companies (20). Since the 1990’s, Corporate Social Responsibility (CSR) has emerged as a strategic approach for companies to create social impact and strengthen ties with stakeholders, including the wider community, which is often realized through corporate volunteering (21, 22). Such CSR initiatives most commonly focus on education, environment, health and welfare, and services for youth and senior citizens (21). In this context, corporate volunteering may offer a valuable way to mobilize additional resources and tap into volunteer potential for mental health care, reaching beyond peers and existing healthcare professionals or volunteers. Recent research indicates that corporate volunteering, particularly when centered on contributing to others, can enhance employees’ sense of meaningfulness in their daily work (23). It also fosters employees’ positive emotions, work engagement, social connections, and self-esteem (24). Promising results were also shown in initiatives such as buddy projects, which pair volunteers with individuals from various populations to enhance mental and physical well-being, benefiting both the populations – by reducing loneliness, strengthening self-reliance, and improving well-being - and the volunteers, who gain meaningful, enjoyable, and skill-building experiences (8, 25, 26).

Despite these promising results, we found no empirical research examining the intersection of corporate employees, social interaction interventions, and older adults with mental illness. While literature on corporate volunteering is extensive (27), the role of companies in geriatric mental health care remains underexplored (28, 29), with little understanding of the barriers and facilitators to participation in social interventions. This study therefore aims to explore factors associated with company employee participation in a walking project developed for psychiatric outpatients aged 60 and older. Our findings may contribute to improving the collaboration between companies and mental health care providers, potentially strengthening informal care networks, improving patient well-being, reducing loneliness, and combating stigma.

## 2 Methods

### 2.1 Design

A qualitative, exploratory design was employed. Companies (profit and nonprofit) were invited to participate in a project connecting outpatients from a mental health institute with their employees, aiming to create sustainable “walking pairs” (patients with employees) for weekly walks lasting approximately 30 minutes to one hour for longer than three months. Walking was chosen as an accessible, low-threshold activity for both patients and employees, supporting routine social engagement and promoting physical and mental health. Through qualitative interviews, this study explored factors associated with employee participation in the project.

### 2.2 Data collection

Data were collected during six months. Companies around Amersfoort, the Netherlands, were identified using an online search tool (*https://bedrijvenopdekaart.nl*), allowing a selection based on size and organizational structure (profit and nonprofit, family-owned and corporate businesses). This ensured a diverse sample in terms of ownership, management styles, and decision-making processes.

There were no specific criteria regarding employee numbers or exclusions. A total of 250 companies were contacted to participate in the interviews, the project, or both. Relevant stakeholders—such as human resources (HR) representatives, vitality managers, or chief executive officers (CEO’s)— were identified through a targeted search in collaboration with the companies, and approached by telephone, email, or online contact form. A flyer supported interview arrangements. Each company was contacted up to two times, using the same or alternative communication channels if no response was received initially.

Semi-structured interviews were conducted and guided by an interview guide covering key topics: e.g. attitudes toward the walking project, factors that could influence participation in the project, and on public stigma related to (older) psychiatric patients. Interviews were audio-recorded, transcribed verbatim, anonymized, and supplemented with fieldnotes. Alternative data collection methods were used when interviewing was not possible. The decision on when data saturation was reached, was based on information gathered from the interviews, as described by Saunders and colleagues (30).

### 2.3 Data analysis

The data analysis process integrated the Constant Comparative Method (31, 32), with a thematic approach (33), aiming to generate sets of themes on the specific interview topics.

Analysis was conducted in six phases: i.e., data preparation, familiarization with the data, finalization of the analysis strategy, open coding, axial coding, and selective coding.

Data preparation involved verbatim transcription of interviews and notes, using Atlas.ti (version 24). During familiarization, two researchers (KK, JS) independently performed open coding, followed by consensus-building on coding strategies. Codes were prefixed according to interview topics to organize broad yet flexible categories of codes. Open coding encompassed assigning descriptive labels to meaningful data units (one word, a few words, a sentence, or more) by two independent coders (KK, JS). Discrepancies were resolved through discussion, with ongoing thematic development through constant comparison.

After coding five interviews, axial coding commenced, grouping codes to discovering themes in the data. Prefixed categories and codes were reviewed to confirm relevance. Subsequently, sub-categories were created within each main category.

Remaining interviews were analyzed, and their codes were integrated into the themes retrieved. Selective coding began at this stage, aiming to integrate and refine complete categories and develop them into themes, revealing the underlying meaning behind groupings.

### 2.4 Ethical procedure

Informed consent was obtained from all participants. To ensure anonymity, code names were assigned and used in documentation, and personal identifiers excluded from transcripts and records. Data was handled in accordance with privacy and confidentiality standards, storage and access limited to authorized personnel only. The protocol was reviewed by the Medical Ethics Committee of Isala Klinieken (case no. 23020) and scientific board of GGz Centraal.

## 3 Findings

A total of 13 out of 250 contacted companies responded (5% response rate). These companies were contacted by telephone (n=6), online contact form (n=5), or email (n=2). Next, 17 respondents from these 13 companies consented to semi-structured interviews, including: three chief executive officers, seven managers (from HR, vitality and office departments), three advisors (in HR and vitality), and four employees. A detailed overview of companies involved in the interviews is provided in **Table 1**. Additional data was collected from 66 companies that declined to participate in an interview and the project, mainly via e-mail and short telephone conversations, and in few cases via an introductory meeting at the respondent’s location or a Microsoft Teams meeting. Reasons for declining are specified in **Table 2**.

**Table 1:** Characteristics of companies and respondents involved in interviews.

Company No.	Branche	Profit status	Ownership	Number of employees	Respondent (Res. no.)
1	Food	Profit	Cooperative	> 1000	Vitality manager (R1)
2	Healthcare	Nonprofit	Board members	< 500	Manager (R3), employees (R4, R5, R6, R7)
3	Information Technology	Profit	Share holders	< 500	HR manager (R8)
4	Social services	Nonprofit	Municipal governance	< 1000	HR manager (R10)
5	Consultancy	Profit	Share holders	< 500	HR advisor (R11)
6	Total installation contractor	Profit	Share holders	< 500	HR advisor (R16)
7	Retail	Profit	Share holders	< 100	Manager (R19)
8	Supply chain technology	Profit	Share holders	< 200	HR manager (R20)
9	Hospitality	Profit	Family	< 50	Chief executive officer (R22)
10	Consultancy	Profit	Share holders	< 50	Office manager (R23)
11	Hospitality	Profit	Share holders	< 50	Chief executive officer (R24)
12	Service provision	Profit	Share holders	< 50	Chief executive officer (R25)
13	Facility management	Profit	Share holders	< 500	HR advisor (R26)

**Table 2:** Reasons for non-participation in interview and project.

Main reason for non-participation	Number of companies
Time constraints	12
Other priorities	11
Staff shortage	8
No interest (reason not specified)	8
Busy with other CSR projects	6
Project not compatible with company practices/policies	6
No further response after initial contact	4
Employees deemed not suitable for project	3
Prefers to contribute to project in other ways	3
Public stigma	2
Reorganization	1
Bankruptcy	1
Company has moved	1

**Table 3** provides an overview of the findings after thematic analysis of both the interviews and additional data (see also paragraphs 3.1 to 3.3). Deeper interpretation of data will be addressed in the discussion.

**Table 3:** Overview of findings.

Main topic	Respondents’ views on related themes
Attitude towards the project (Paragraph 3.1)	Positive attitude: Enthusiasm, recognized benefits (reduced sedentary behavior of employees, reduced patient loneliness and improved QoL, health benefits, employee empathy development), tangible, stimulating, importance of mental health care. Improvement of patient perception, increased interest in patient well-being
Associated factors (Paragraph 3.2)	Views on volunteer work (3.2.1.): Importance: Valuable, enjoyable and enriching for employees. Complexity of contributing to volunteer work: Often ‘one-time’ actions, diverging opinions on volunteer projects as social responsibility versus private domain, economic value versus social value Preconditions (3.2.2.): Integrating participation during work hours, communication (success stories, clarifying time commitment, clear frameworks and explanations, accessible information, publicity strategies), motivators and rewards, discouraging mandatory participation Employee-related factors (3.2.3.): Anticipation of modest registration rates and limited commitment, intrinsic motivation is pivotal, personal circumstances and life stages influence participation, social employee characteristics foster participation, employee benefits play a significant role Patient-related factors (3.2.4.): Matching participants, patient enjoyment, effective communication, target demographic understanding Company related factors and suggestions for implementation in companies (3.2.5.): Corporate Social Responsibility (CSR), complex agendas of higher management, company processes, organizational type and size, output takes precedence over participation, suggestions for implementation (HR department involvement, start with a pilot project, policies promoting volunteering, align with office employee presence and their walking habits, responsibility for project initiation, collaboration with municipalities
Public stigma related to (older) psychiatric patients (Paragraph 3.3)	Perceptions of patients (unjustified stigmas, media portrayal, respondents hold varied personal perceptions), opinions vary regarding the impact of stigma

### 3.1 Attitude towards the project

Overall, respondents view the project as meaningful and engaging, with enthusiasm and willingness to participate. They think of the project as practical and concrete, but also stimulating and educational. Respondents anticipated potential benefits for both employees and outpatients, which they believe outweigh company investment. Two key issues stood out: 1) encouraging sedentary office staff to become more physically active, and 2) helping reduce patient loneliness. A respondent notes:

*“I can imagine, it cuts both ways [ … ] that combination, I find it interesting.”* R10

Walking is seen as healthy and beneficial for mental health, offering employees an opportunity to take breaks, stay active, and boost both vitality and job satisfaction. The project was also valued for offering tailored solutions to a broad societal issue, enhancing patients’ quality of life:

*“I follow the news as well [ … ] people just don’t know where to turn, which institution to go to [ … ] it’s not all tailored, of course, but the fact that there’s nowhere to go is really heartbreaking. This is simply a good initiative, to be able to offer something in that area.”* R25

Another respondent emphasized the importance of mental healthcare, sharing a personal connection:

*“I’ve had personal experience with mental health services in the past [ … ] I find mental health care extremely important. I know how valuable it is.”* R19

Respondents anticipated the project will raise awareness of patient well-being among employees and expected collaborative walks will lead to a more positive perception of patients:

“*I think there are quite unjustified stigmas, and such a walk can really help with that.”* R8

### 3.2 Influencing factors for participation in the project

Five subthemes for participation in the project were identified: 1) Views on volunteer work, 2) Preconditions, 3) Employee-related factors, 4) Patient-related factors and the impact of stigma, 5) Company-related factors and suggestions for implementation in companies.

#### 3.2.1 Views on volunteer work

Volunteer work is perceived as valuable and enjoyable for participating employees. It is seen as something that can make employees’ work worthwhile:

*“People find it very enriching to contact a target group in this way, one they normally do not interact with.”* R11

According to respondents, initiatives regarding voluntary work within companies often concern single, one-time actions (i.e., without repetition) that are not necessarily work-related:

*“If employees participate in a project, it’s often for half a day. For example, working together to restore a playground just around the corner, because that makes it fun and enjoyable for everyone.”* R1

Implementing structural volunteer work is complex. Some companies consider it a social responsibility, others a personal one. Management’s willingness to invest is doubted:

*“It depends on whether they believe it’s important for the company and the employees.”* R1

Moreover, economic value is more dominant for companies and their employees as compared to social value:

*“People are driven by what they produce at work, because they are judged on that by the manager. It would be beneficial if we started judging people on what they contribute to society. [ … ] I believe that when it comes to human sustainability and rewarding ‘doing good for others or society’, we still have a long way to go in that regard.”* R1

Also, informal caregiving, while essential, seems not widely supported in the Netherlands:

*“You see a lot of differences around the world [ … ] Especially in Asia. Taking care of elderly people in general [ … ] there, it’s almost strange if you wouldn’t do it. While here it’s seen as a burden: ‘Oh, you’re also a caregiver?’”* R1

#### 3.2.2 Preconditions

Integrating participation during working hours is preferred, with breaks ideal for walking. Clear frameworks outlining objectives, context, benefits, obligations, and expectations are crucial for justifying employees’ time commitment and maintaining productivity.

*“I think it’s important that you clearly explain why you are doing this and why it’s important. What’s the underlying idea from your vision? [ … ] But also ‘What’s in it for them?’”* R10

Raising project awareness is challenging, with success stories as key tools. Clear, accessible information online or via the company’s intranet is crucial. However, opinions on publicity vary among respondents; some prefer campaigns, others consider word-of-mouth sufficient. While Corporate Social Responsibility (CSR) benefits company image, respondents warn against overt display:

*“If it becomes some sort of showpiece [ … ] nobody is waiting for that. I think it would also be at the expense of the patient.”* R10

Respondents discourage mandatory participation, fearing it negatively impacts employee motivation. Five of seventeen respondents suggest compensation or rewards as motivators for participation. One disagrees:

*“Our people won’t engage in it solely for monetary reasons [ … ] the additional people you attract by attaching money to it, I think it makes hardly any difference.”* R11

#### 3.2.3 Employee-related factors

Some managers and HR advisors struggle to gauge employees’ attitudes towards participation, expecting modest registration rates and limited commitment:

*“This is not an initiative where a hundred and fifty people sign up at once.”* R8

Employees’ intrinsic motivation is pivotal for participation, driven by their natural inclination, interests beyond work, and their concern for social impact. Personal circumstances, family commitment or leisure activities may take precedence:

*“I believe that participating in activities outside of work will be quite challenging, employees are often already contributing to the social environment—for instance, serving as a trainer for the local football club or engaging in various school-related activities.”* R1

Respondents emphasize traits like sociability, curiosity, communication skills, and openness to new experiences are crucial for engaging employees with outpatients. Whereas involvement of employees with neurodiversity traits may be more challenging:

*“A selective group would qualify for pairing with mental health care patients, because some of the employees have enough to deal with themselves. Possibly due to characteristics of autism or ADHD.”* R16

Employee benefits matter, like breaks and reduced workload due to walks. However, diverse break preferences hinder walking, as some opt to continue working or stay indoors.

*“The act of walking itself is already an adventure, isn’t it? [ … ] It’s already a challenge to get employees outside during their break.”* R8

Respondents expected that healthcare workers and staff members in social services prefer walking without clients:

*“I can imagine that, because our staff also has to deal with people with disabilities [ … ] they also like to take a walk, but just by themselves or with a colleague.”* R10

#### 3.2.4 Patient-related factors

Respondents highlight that walking with a stranger can be uncomfortable for both employees and patients. They advocate for matching based on shared interests:

*“When an employee comes along who has the same hobby or has had that hobby before, or shares the same interest and can talk a lot about it, there is an instant connection.”* R3

Respondents stress patient enjoyment and communication ability are key. Employee engagement requires insight into the target demographic, necessitating a profile description of older mental health patients served by the outpatient clinic:

*“I think it’s important to clearly explain the target group. Otherwise, a certain image might be created [ … ] People in residential care are often seen as quite strange. If that’s the perception employees have, I can imagine they might be put off by it. If the outpatient group is different from people in residential care, I think it’s important to explain that as well.”* R10

#### 3.2.5 Company related factors and suggestions for implementation in companies

Respondents value CSR, but companies face challenges in fully embracing it due to complex management agendas and the involvement of multiple stakeholders. Project participation requires navigating company processes and decision points, securing leadership support, and appointing a dedicated contact person to endorse the initiative:

*“Various directors of all advisory groups can have an opinion about it and if they all think it’s a great initiative and that employees spend time on this from work time [ … ] I have to create a bit of ‘buy-in’ for that.”* R1

In an interview with a CEO, no process was needed; the decision was made directly:

*“It is an important contribution you can make. I would be more than willing to facilitate when an employee expresses a desire to participate.”* R25

Organization type and size affect participation enthusiasm. Three HR advisors stress work priorities, noting cutbacks and staff shortages leave no time for the project:

*“I think I would rather use that time for our own employees, because they are our main priority, right? Rather than investing time in clients of mental health care.”* R10

Regarding project organization, proper presentation and HR involvement are emphasized for project viability. Respondents pointed out the complexity of implementation and suggest pilot projects with select employees, facilitating decision-making within companies:

*“Therefore, we often work with pilots [ … ] because they are reversible, making it somewhat easier, requiring less senior approval to make a particular decision.”* R1

Policies on ‘sustainable employability’ or volunteering during work hours are emphasized by respondents. They suggest shared responsibility between companies and the mental health institute, and collaboration with the municipality. Feasibility depends on office presence and walking habits.

### 3.3 Public stigma related to (older) psychiatric patients

When discussing mental health patients, respondents used general terms such as ‘someone like that’, ‘that kind of patient’, or ‘those people’. They hold varying perceptions, ranging from aggressive stereotypes—labelling patients as ‘crazy’ and ‘unpredictable’—to nuanced, stigma-free understandings and, also highlight unjustified stigmas:

*“Many reports in the media about mental health care aren’t positive. It’s all about excitement and danger [ … ] Because the media only covers psychiatric patients in forensic care, or people who suddenly set things on fire or harm others. That’s not average (laughs).”* R1

Opinions vary on stigma’s impact on participation. Some respondents believe mental health is irrelevant, others view it as a barrier:

*“There’s the task of getting them to walk with someone they may not know well and who requires some extra attention.”* R8

## 4 Discussion

This study aimed to explore factors associated with company employee participation in a waking project involving older psychiatric outpatients. Our exploratory method allowed us to uncover these factors, which we can now interpret in the light of existing theory on public stigma and CSR. Respondents generally viewed the project positively, emphasizing benefits for both employee well-being and patient support. This favorable outlook highlights the potential of targeted interventions in elderly mental health care.

However, significant barriers to structural volunteer engagement within companies were identified, complementing literature on the complexity of implementing volunteer work (28). The low (5%) response rate from contacted companies reflects a significant challenge of securing company engagement for ongoing volunteer efforts. This reluctance may reflect broader issues around stigma and discomfort with mental illness, as identified in the interviews. Franjić (34) noted that stigma, rooted in ignorance and fear, hinders social inclusion and engagement of people with mental illness.

Respondents expressed walking with psychiatric patients may evoke discomfort. Companies might hesitate to support initiatives requiring sustained interaction with this group. As respondents highlighted, media portrayals often exaggerate negative aspects of mental illness, creating a skewed perception that may deter employees from engaging with patients. This reflects research on the unjustified perception of individuals with mental illness, often stereotyped as dangerous (34, 35). Respondents with personal or professional experience with mental illness or disabilities showed greater openness towards patients. Recent study found healthcare professionals with close ties to mental illness, exhibited lower levels of stigma (36). Interestingly, health- and social care companies were somewhat reluctant to participate, as respondents expected employees to prefer walking without clients.

A potential barrier lies in respondents’ expectation that patients will demonstrate enjoyment. In a recent study, volunteers assumed that patients would exhibit enthusiasm during project involvement (37). Yet, older patients may experience symptoms such as anhedonia, limiting social engagement and potentially impacting project success (38). Although research shows mental health patients are often genuinely motivated to join physical activities, psychiatric barriers may limit actual participation (39–41). Nevertheless, respondents believed the project could gradually shift employee perspectives and reduce stigma, fostering greater empathy for individuals with mental health issues. They stressed the importance of providing a clear profile of the target demographic in advance, supporting findings from a study in which volunteer training covered communication, confidentiality, boundaries, risk assessment and emergencies to prepare volunteers for working with psychiatric patients (37).

Practical and organizational barriers included difficulties integrating the project into workflows, limited senior management support, and corporate focus on output over social engagement. Participation also should not conflict with work responsibilities, which makes companies reluctant to allocate resources to non-core activities. This points to the need for strategies to increase corporate buy-in, such as aligning volunteer projects with CSR objectives and clearly communicating mutual benefits for employees and organizations.

Leadership proved pivotal in enabling social initiatives: one CEO endorses the initiative, linking it to broader societal concerns. CSR research among executives (42) confirms such decisions often stem from personal beliefs and priorities.

### 4.1 Recommendations for practice

Based on the results, five practical recommendations for implementing physical activity initiatives, connecting employees of commercial companies and mental health patients, are outlined below.

#### 4.1.1 Engage companies that value corporate social responsibility

A structured approach to expectation management and support is essential. Communicate the benefits of participation, and highlight how initiatives align with CSR goals to secure management buy-in. Collaboration with HR and key stakeholders may enable implementation. Companies lacking resources may seek municipal partnerships for support and funding.

#### 4.1.2 Engage enthusiastic, intrinsically motivated employees

Encourage participation from motivated employees by ensuring they understand the project’s objectives, benefits and expectations. Match employees with patients who share similar interests and provide support, incentives, and opportunities for professional development. Consider various employee schedules to facilitate participation.

#### 4.1.3 Provide education and support for employees

Offer sessions led by mental health professionals to address stigma, educate employees on patient conditions, and effective interaction techniques. A dedicated contact person should be available throughout the project to provide support.

#### 4.1.4 Start with a pilot

Implement a pilot program with a select group of enthusiastic employees. Use this to test, refine, and adjust the project before scaling up the initiative company-wide.

#### 4.1.5 Utilize promotional strategies

Raise awareness through success stories, informational sessions, and accessible online resources. Tailor messaging to highlight mutual benefits for employees and patients to boost engagement and interest.

### 4.2 Strengths and limitations

This study presents significant findings supported by a multidisciplinary research team, contributing to a robust and well-rounded approach to data collection and -analysis, facilitating comprehensive insights and enhancing the study’s relevance in both academic and practical contexts.

Certain limitations must be taken into account when interpreting the findings. First, a limitation is that, despite extensive outreach to 250 companies, only 13 participated in the interviews. The low response rate may have introduced selection bias if participating companies differed systematically from non-respondents, which not only limits the generalizability of the finding but may also influence the credibility of the identified themes. Second, demographic information was not systematically collected. This prevents analysis of how such factors may have influenced participants’ perspectives and limits the transferability of the findings. Future research should consider including these data for a more nuanced interpretation. Third, due to the exploratory nature of the study and the small, unbalanced sample (2 of 13 companies were non-profit), findings were not stratified by organization type or size. Future research with a larger and more diverse sample could investigate these factors for more nuanced insights. Finally, stigma was identified as a putative barrier. We hypothesized that walking in pairs would reduce stigma, associated with psychiatric patients. Future research, aiming to explore the impact of walking in pairs, should incorporate pre- and post-assessment of stigma, and other measures such as loneliness, or physical health, depending on the scope of the study.

## 5 Conclusion

This study explored factors influencing employee participation in a walking initiative for older, psychiatric outpatients. While the study highlights the positive impact of mental health volunteer initiatives on both employees and patients, it also emphasizes the need for greater company involvement in such initiatives. By addressing barriers like stigma and discomfort, and creating a supportive environment that encourages employee participation, companies can play a pivotal role in enhancing both employee well-being and patient care, ultimately contributing to a more inclusive and compassionate society.

## Data Availability

The original contributions presented in the study are included in the article/supplementary material, further inquiries can be directed to the corresponding author/s.
